# Effects of Combined Aspirin and Clopidogrel Therapy on Cardiovascular Outcomes: A Systematic Review and Meta-Analysis

**DOI:** 10.1371/journal.pone.0031642

**Published:** 2012-02-13

**Authors:** Yu-Hao Zhou, Xin Wei, Jian Lu, Xiao-Fei Ye, Mei-Jing Wu, Jin-Fang Xu, Ying-Yi Qin, Jia He

**Affiliations:** 1 Department of Health Statistics, Second Military Medical University, Shanghai, China; 2 School of Medicine, Shanghai Jiao Tong University, Shanghai, China; University of British Columbia, Canada

## Abstract

**Background:**

Aspirin and clopidogrel monotherapies are effective treatments for preventing vascular disease. However, new evidence has emerged regarding the use of combined aspirin and clopidogrel therapy to prevent cardiovascular events. We therefore performed a comprehensive systematic review and meta-analysis to evaluate the benefits and harms of combined aspirin and clopidogrel therapy on major cardiovascular outcomes.

**Methodology/Principal Findings:**

We systematically searched Medline, Embase, the Cochrane Central Register of Controlled Trials, reference lists of articles, and proceedings of major meetings to identify studies to fit our analysis. Eligible studies were randomized controlled trials assessing the effect of combined aspirin and clopidogrel therapy compared with aspirin or clopidogrel monotherapy. We identified 7 trials providing data with a total of 48248 patients. These studies reported 5134 major cardiovascular events, 1626 myocardial infarctions, 1927 strokes, and 1147 major bleeding events. Overall, the addition of aspirin to clopidogrel therapy as compared to single drug therapy resulted in a 9% RR reduction (95%CI, 2 to 17) in major cardiovascular events, 14% RR reduction (95%CI, 3 to 24) in myocardial infarction, 16% RR reduction (95%CI, 1 to 28) in stroke, and 62% RR increase (95%CI, 26 to 108) in major bleeding events. We also present the data as ARR to explore net value as the reduction in cardiovascular events. Overall, we observed that combined therapy yielded 1.06% decrease (95%CI, 0.23% to 1.99%) in major cardiovascular events and 1.23% increase (95%CI, 0.52% to 2.14%) in major bleeding events.

**Conclusion/Significance:**

Although the addition of aspirin to clopidogrel resulted in small relative reductions in major cardiovascular events, myocardial infarction, and stroke, it also resulted in a relative increase in major bleeding events. In absolute terms the benefits of combined therapy, a 1.06% reduction in major cardiovascular events, does not outweigh the harms, a 1.23% increase in major bleeding events.

## Introduction

Cardiovascular disease is the leading cause of premature morbidity and mortality worldwide for both men and women [1], accounting for 30.9% of global mortality and 10.3% of the global burden of disease [2]. Antiplatelet therapy, which is recommended as the standard practice [3], can reduce the harm of cardiovascular disease in patients with peripheral arterial disease. However, its effects in patients with cardiovascular harm factors remain unclear. Over the past decades, several studies [4]–[6] have demonstrated that aspirin is effective in the prevention of cardiovascular events, and long-term aspirin therapy reduces the annual harm of serious vascular disease by approximately 25% [7]. Recently, clopidogrel (Plavix; Sanofi-Aventis and Bristol-Myers Squibb) has emerged as a new drug commonly used for the secondary prevention of cardiovascular disease, and evidence [8] shows that clopidogrel therapy is superior to aspirin for the secondary prevention of serious vascular events. However, in many cases, aspirin or clopidogrel therapy alone is not sufficient to prevent ischemic events in patients at high harm [9]. It is necessary to develop additional effective preventive therapies.

The goals of prevention for cardiovascular events are anti-thrombus and platelet aggregation, for which both aspirin and clopidogrel are promising. Clinical efficacy of these drugs for reducing serious vascular events and mortality in patients with high harm has been shown in several large-scale clinical trials [10], [11]. However, whether dual antiplatelet therapy with aspirin plus clopidogrel is more effective than a single antiplatelet agent in patients with high harm remains uncertain.

Recently, several large-scale randomized controlled trials investigating the use of aspirin combined with clopidogrel therapy have been performed [12]–[14]. Some trials indicated that combined therapy had beneficial effects, while others showed little effect and some studies even found that combined therapy could induce drug-related adverse reactions, such as bleeding events. This leads to uncertainty regarding the cardiovascular protective effects of combined therapy, which makes interpretation of the results difficult for clinicians. To better understand the effect of combined therapy on cardiovascular outcomes, data from these recent trials needed to be evaluated to formulate a conclusion regarding the efficacy of combined therapy. We therefore conducted a systematic review and meta-analysis of pooled data from randomized controlled trials, including the latest evidence of the association of aspirin plus clopidogrel therapy on the harm of serious vascular events and any possible adverse reactions in patients with high cardiovascular harm factors.

## Methods

### Data sources, search strategy, and selection criteria

We gathered data from randomized controlled trials to assess the effect of combined aspirin with clopidogrel therapy on the harm of cardiovascular outcomes. To be consistent with other large-scale meta-analysis protocols, we included trials comparing combined therapy with a control, excluding any studies with a follow-up period of less than 12 months, in order to alleviate systematic error and resultant bias, and ensure the reliability of our conclusion.

Randomized controlled trials and literature reporting trials of combined therapy in English met the eligibility criteria for our meta-analysis, regardless of publication status (published, unpublished, in press or in progress). Relevant trials were identified with the following procedure:

1. Electronic searches: We searched PubMed, EmBase, and the Cochrane Central Register of Controlled Trials with a date up to March 20, 2011. We use the terms of “aspirin” OR “clopidogrel” AND “clinical trial”.
2. Other sources: We contacted authors to obtain any possible additional published or unpublished data and searched the proceedings of the annual meeting in the Cochrane Cardiovascular Disease Group Specialized Register. Furthermore, we searched ongoing randomized controlled trials in the metaRegister of Controlled Trials, which lists trials that are registered as completed but not yet published. In addition, we reviewed bibliographies of publications for potentially relevant articles. Medical subject headings, methods, patient populations, interventions and outcome variables of these trials were used. This review was conducted and reported according to the PRISMA (Preferred Reporting Items for Systematic Reviews and Meta-Analysis) Statement issued in 2009 (Checklist S1) [15].

All included trials evaluated the development of serious vascular events after treatment with active or control therapy. In addition, we excluded all studies with less than 100 patients and less than 12-month follow-up. Further, identified trials should report on one or more of the following primary or secondary outcomes: major cardiovascular events, myocardial infarction, stroke, mortality, major bleeding events, and other possible adverse reaction.

### Data collection and quality assessment

The identified 7,038 studies were reviewed by 2 authors (Xin Wei and Mei-Jing. Wu) independently. Other two reviewers (Jin-Fang. Xu and Jian Lu) independently checked each full-text trial for eligibility and extracted and tabulated all relevant data with a standardized flow path. Extracted data included the year of publication, the number of patients enrolled, interventions, primary or secondary prevention protocols, cardiovascular harm factors, the duration of follow-up, and outcome events. Disagreements regarding the data were settled by consensus between all authors. Additional tabular data were retrieved by direct contact with the corresponding authors and discrepancies in the data were corrected when a consensus was reached. Study quality was assessed using the Jadad score [16] (Yu-Hao. Zhou) on randomization, concealment of the treatment allocation, blinding, completeness of follow-up, and the use of intention-to-treat analysis.

### Statistical analysis

We allocated the results of each randomized controlled trial as dichotomous frequency data. Individual study relative risks (RRs) and 95% confidence intervals (CIs) were calculated from event numbers extracted from each trial before data pooling. The overall relative risk (RR) with 95% CIs of serious vascular events and any possible adverse reaction was also calculated. Although the fixed-effect and random-effects models yielded similar conclusions, we chose the random-effects model with Mantel-Haenszel statistics for the meta-analysis, which assumed that the true underlying effect varies among included trials. Moreover, many investigators also consider the random-effects model to be a more natural choice than the fixed-effect model in medical decision-making contexts [17], [18]. The variability, expressed in percentage, across studies attributable to heterogeneity beyond chance was estimated with the I^2^ statistic [19]. Egger's test [20] was used to check for potential publication bias. We explored potential heterogeneity in estimates of treatment effect with univariate meta-regression. We also performed subgroup analysis by mean age, follow-up years, and study quality. All reported P values were two-sided and P values of less than 0.05 were regarded as significant for all included studies. Statistical analyses were carried out using STATA (version 10.0).

## Results

**Figure 1:**
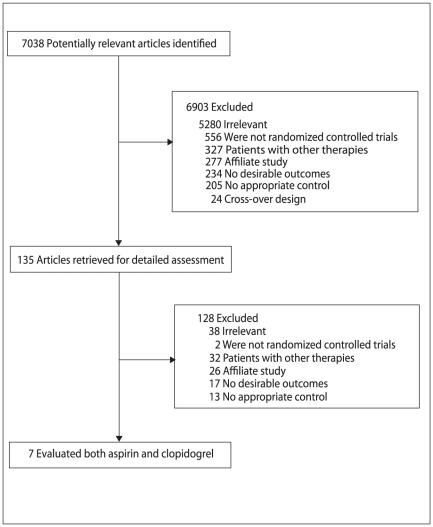
Flow diagram of the literature search and trials selection process.

We identified 7,038 potential studies from our systematic review, and 6,903 of them were excluded after a preliminary review of searches. The remaining 135 studies were retrieved for detailed assessment. Of these, 7 randomized controlled trials met our inclusion criteria (Figure 1 and Protocol S1 [15]). 6 of these evaluated combined therapy comparing with aspirin therapy alone [9], [12]–[14], [21], [22] and the other one study [23] evaluated combined therapy comparing with clopidogrel alone. The 7 trials provided data of 48,248 patients (mean, 6,893 patients and range from 113 to 15,603). Table 1 summarizes the baseline characteristics of the included studies and their participants. The primary outcomes were major cardiovascular events available in 6 trials, myocardial infarction in 7 trials, stroke in 7 trials, total mortality in 6 trials, vascular death in 4 trials, nonvascular death in 4 trials, revascularization in 2 trials, and major bleeding in 6 trials. Although the included trials scarcely reported on the key indicators of trial quality, the quality of the trials was also assessed according to the pre-fixed criteria using the Jadad score. Overall, two trials scored 5, two trials scored 4, and the remaining three trials scored 3.

**Table 1:** Design and characteristic of trials included in the systematic review and meta-analysis.

Source	No. of patients	Mean age, y	Sex (male)	Inclusion criteria	Primary/secondary prevention	Intervention	Duration of follow-up (months)	Jaded score
The CURE Investigators [9]	12562	64.2	7726 (61.5%)	acute coronary syndromes without ST-segment elevation	secondary	(1)Clopidogrel (75 mg daily) plus Aspirin (75 to 325 mg daily) (2)Aspirin (75 to 325 mg daily)	12	4
SJ Park 2010 [12]	2701	62.0	1883 (69.7%)	Stents used >12 month	secondary	(1)Clopidogrel (75 mg daily) plus Aspirin (100 to 200 mg daily) (2) Aspirin (100 to 200 mg daily)	19.2	3
The ACTIVE Investigators [13]	7554	71.0	4397 (58.2%)	atrial fibrillation	secondary	(1)Clopidogrel (75 mg daily) plus Aspirin (75 to 100 mg daily) (2) Aspirin (75 to 100 mg daily)	43.2	4
CASCADE Trial [14]	113	66.5	101 (89.4%)	coronary artery bypass grafting	secondary	(1)Clopidogrel (75 mg daily) plus Aspirin (162 mg daily) (2) Aspirin (162 mg daily)	12	5
CHARISMA Investigators [21]	15603	64.0	10959 (70.2%)	multiple atherothrombotic risk factors	secondary	(1)Clopidogrel (75 mg daily) plus Aspirin (75 to 162 mg daily) (2) Aspirin (75 to 162 mg daily)	28	3
CREDO Investigators [22]	2116	61.7	1510 (71.4%)	Privious PCI	secondary	(1)Clopidogrel (75 mg daily) plus Aspirin (81 to 325 mg daily) (2) Aspirin (81 to 325 mg daily)	12	5
MATCH investigators [23]	7599	66.3	4778 (62.9%)	at least one vascular risk factor	secondary	(1)Clopidogrel (75 mg daily) plus Aspirin (75 mg daily) (2) Clopidogrel (75 mg daily)	18	3

**Figure 2:**
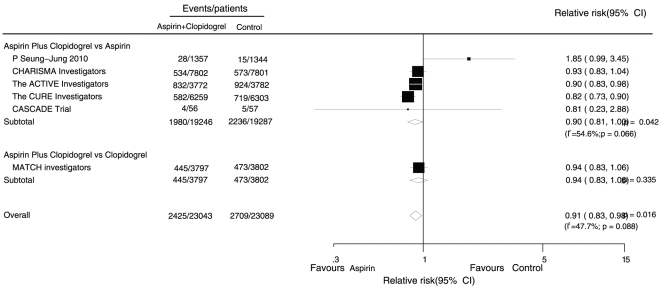
Effects of aspirin with clopidogrel therapy on risk of major cardiovascular events.

Data for the effect of combined aspirin and clopidogrel therapy on major cardiovascular events were available from 6 trials, which included 46,132 patients and reported 5,134 serious vascular events (Figure 2). Overall, we observed that with combined therapy, the harm of major cardiovascular events was significantly reduced by 9% (RR, 0.91; 95% CI, 0.83 to 0.98) compared with single drug therapy.

**Figure 3:**
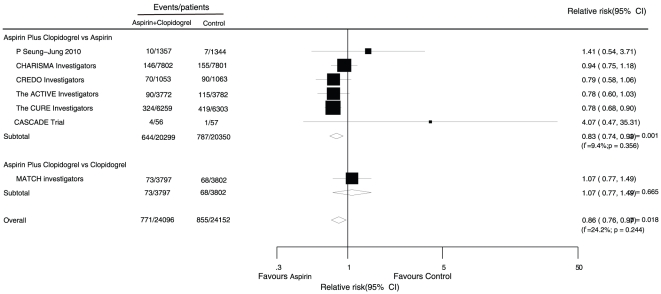
Effects of aspirin with clopidogrel therapy on risk of myocardial infarction.

Data for the effect of aspirin combined with clopidogrel therapy on myocardial infarction were available from 7 trials, including 48,248 patients and 1,626 events of myocardial infarction (Figure 3).

Combined therapy resulted in a 14% reduction in the harm of myocardial infarction compared with single drug therapy (RR, 0.86; 95% CI, 0.76 to 0.97).

**Figure 4:**
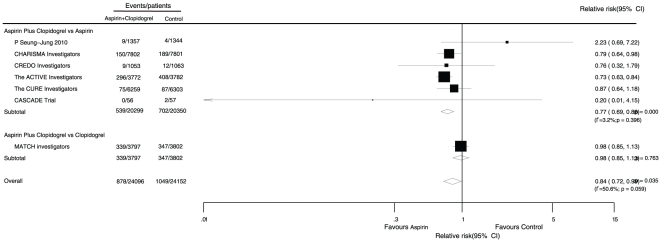
Effects of aspirin with clopidogrel therapy on risk of stroke.

Data for the effect of aspirin combined with clopidogrel therapy on stroke were available from 7 trials, which included 48,248 patients and reported 1,927 stroke events (Figure 4). Overall, combined therapy reduced the harm of stroke by 16% when compared with single drug therapy (RR, 0.84; 95% CI, 0.72 to 0.99).

Six trials reported the effect of aspirin combined with clopidogrel therapy on total mortality, which including 35,686 patients and recorded 2,889 deaths (Table 2). Of the included trials, 4 trials reported separate data for vascular death (27,828 patients and 2,108 vascular deaths) and 4 trials provided separate data for nonvascular death (27,828 patients and 710 nonvascular deaths). Overall, there was no evidence to show that combined therapy could reduce the risk of mortality, regardless of total mortality, vascular death, or non-vascular death.

**Table 2:** Evidence profile for Aspirin and Clopidogrel on the risk of vascular events.

		Intervention group (events/total patients)	Control group (events/total patients)	RR (95% CI)	P value	I^2^ (%)	P value for heterogeneity
Major cardiovascular events	Aspirin with clopidogrel vs aspirin alone	1980/19246	2236/19287	0.90 (0.81, 1.00)	0.04	55	0.07
	Aspirin with clopidogrel vs clopidogrel	445/3797	473/3802	0.94 (0.83, 1.06)	0.33	-	-
MI	Aspirin with clopidogrel vs aspirin alone	644/20299	787/20350	0.83 (0.74, 0.93)	0.001	9	0.36
	Aspirin with clopidogrel vs clopidogrel	73/3797	68/3802	1.07 (0.77, 1.49)	0.67	-	-
Stroke	Aspirin with clopidogrel vs aspirin alone	539/20299	702/20350	0.77 (0.69, 0.86)	<0.001	3	0.40
	Aspirin with clopidogrel vs clopidogrel	339/3797	347/3802	0.98 (0.85, 1.13)	0.76	-	-
Total mortality	Aspirin with clopidogrel vs aspirin alone	1234/14040	1253/14047	0.99 (0.92, 1.06)	0.70	0	0.61
	Aspirin with clopidogrel vs clopidogrel	201/3797	201/3802	1.00 (0.83, 1.21)	0.99	-	-
Vascular death	Aspirin with clopidogrel vs aspirin alone	918/10087	945/10142	0.98 (0.90, 1.0 6)	0.61	0	0.56
	Aspirin with clopidogrel vs clopidogrel	124/3797	121/3802	1.03 (0.80, 1.31)	0.84	-	-
nonvascular death	Aspirin with clopidogrel vs aspirin alone	266/10087	287/10142	0.93 (0.79, 1.09)	0.38	0	0.95
	Aspirin with clopidogrel vs clopidogrel	77/3797	80/3802	0.96 (0.71, 1.31)	0.82	-	-
revascularization	Aspirin with clopidogrel vs aspirin alone	175/2410	170/2407	1.07 (0.79, 1.44)	0.65	34	0.22
Major bleeding	Aspirin with clopidogrel vs aspirin alone	616/19246	436/19287	1.42 (1.26, 1.60)	<0.001	0	0.64
	Aspirin with clopidogrel vs clopidogrel	73/3759	22/3781	3.34 (2.08, 5.36)	<0.001	-	-

The effect of aspirin combined with clopidogrel therapy on the harm of revascularization was reported in 2 trials, which included 4,817 patients and recorded 345 revascularization events. Overall, there was no effect of combined therapy on the harm of revascularization events compared with single drug therapy (Table 2).

**Figure 5:**
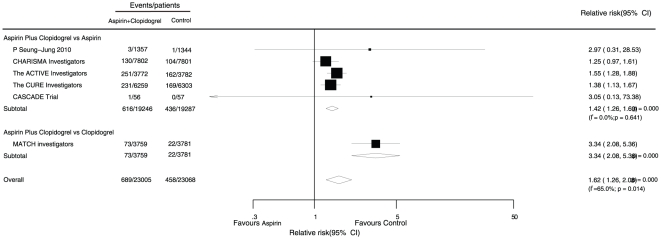
Effects of aspirin with clopidogrel therapy on risk of major bleeding events.

We observed that the primary adverse outcome was major bleeding and this outcome was record by 7 trials (46,073 participants and 1,147 major bleeding events, Figure 5). Overall, combined therapy increased the harm of major bleeding events by 62% when compared with single drug therapy (RR, 1.62; 95% CI, 1.26 to 2.08).

Although combined therapy present beneficial effect than single drug therapy in reducing the harm of major cardiovascular events, myocardial infarction, or stroke, it also significantly increased the harm of major bleeding events. Therefore, we have present the data as absolute risk reduction (ARR) and its confidence intervals to explore net value as the reduction in cardiovascular events, which weighted against any increase in major bleeding events. Overall, the outcome measuring benefit is total cardiovascular events, the pooled ARR showed 1.06% on major cardiovascular events, and 95% confidence intervals ranged from 0.23% to 1.99%. Similarly, the outcome measuring harm comes from major bleeding events, and the pooled ARR showed 1.23%, 95% confidence intervals ranged from 0.52% to 2.14%, we noted that the benefits ARR was not associated with a statistically significant difference compared with harms ARR, which indicated that the benefits do not outweigh the harms for combined therapy.

We noted evidence of heterogeneity in the magnitude of the effect across the included trials for major cardiovascular events, myocardial infarction, stroke, and major bleeding. Therefore, we performed a subgroup analysis to minimize the consequences of heterogeneity among the included trials based on control drugs (Table 2). In addition, we also performed a subgroup analysis for combined therapy compared with different drug therapy based on mean age, follow-up years, and study quality (Table 3). Overall, aspirin combined with clopidogrel therapy was not associated with a reduction in the harm of major cardiovascular events compared with aspirin therapy alone when mean age of included trials was less than 65, follow-up was less than 24 months, and the Jadad score was less than 4. The harm of myocardial infarction with combined therapy was comparative to that with aspirin therapy alone when mean age of the included trials was more than 65, follow-up was more than 24 months, and the Jadad score was less than 4. Similarly, the harm of stroke was not significantly reduced when follow-up of the included trials was less than 24 months and the Jadad score was less than 4. Further, the harm of major bleeding events was ineffectively reduced when the Jadad score of the included trials was less than 4.

**Table 3:** Subgroup analysis of major cardiovascular events, myocardial infarction, stroke and major bleeding events after treatment with aspirin and clopidogrel agents.

		Subgroup	Intervention group	Control group	RR(95% CI)	P value	P value for heterogeneity
**Major cardiovascular events**	**Aspirin with clopidogrel vs aspirin alone**	**Mean age**					
		>65	836/3828	929/3839	0.90 (0.83, 0.98)	0.01	0.87
		<65	1144/15418	1307/15448	0.93 (0.76, 1.13)	0.46	0.01
		**Follow-up (month)**					
		>24	1366/11574	1497/11583	0.91 (0.85, 0.98)	0.007	0.66
		<24	614/7672	739/7704	1.07 (0.58, 1.97)	0.82	0.04
		**Jadad score**					
		4 or 5	1418/10087	1648/10142	0.87 (0.80, 0.93)	0.0001	0.31
		<4	562/9159	588/9145	1.22 (0.63, 2.36)	0.55	0.03
	**Aspirin with clopidogrel vs clopidogrel**	**none**	445/3797	473/3802	0.94 (0.83, 1.06)	0.33	-
**MI**	**Aspirin with clopidogrel vs aspirin alone**	**Mean age**					
		>65	94/3828	116/3839	1.24 (0.29, 5.31)	0.77	0.14
		<65	550/16471	671/16511	0.83 (0.73, 0.94)	0.002	0.35
		**Follow-up (month)**					
		>24	236/11574	270/11583	0.87 (0.73, 1.04)	0.13	0.31
		<24	408/8725	517/8767	0.81 (0.67, 0.97)	0.03	0.30
		**Jadad score**					
		4 or 5	488/11140	625/11205	0.78 (0.70, 0.88)	<0.0001	0.52
		<4	156/9159	162/9145	0.96 (0.77, 1.20)	0.73	0.42
	**Aspirin with clopidogrel vs clopidogrel**	**none**	73/3797	68/3802	1.07 (0.77, 1.49)	0.67	-
**Stroke**	**Aspirin with clopidogrel vs aspirin alone**	**Mean age**					
		>65	296/3828	410/3839	0.73 (0.63, 0.84)	<0.0001	0.41
		<65	243/16471	292/16511	0.83 (0.70, 0.99)	0.03	0.39
		**Follow-up (month)**					
		>24	446/11574	597/11583	0.75 (0.66, 0.84)	<0.0001	0.50
		<24	93/8725	105/8767	0.91 (0.62, 1.33)	0.62	0.33
		**Jadad score**					
		4 or 5	380/11140	509/11205	0.75 (0.66, 0.85)	<0.0001	0.62
		<4	159/9159	193/9145	1.12 (0.43, 2.93)	0.81	0.09
	**Aspirin with clopidogrel vs clopidogrel**	**none**	339/3797	347/3802	0.98 (0.85, 1.13)	0.76	-
**Major bleeding events**	**Aspirin with clopidogrel vs aspirin alone**	**Mean age**					
		>65	252/3828	162/3839	1.56 (1.29, 1.89)	<0.0001	0.68
		<65	364/15418	274/15448	1.33 (1.14, 1.56)	0.0003	0.66
		**Follow-up (month)**					
		>24	381/11574	266/11583	1.42 (1.15, 1.75)	0.001	0.18
		<24	235/7672	170/7704	1.39 (1.14, 1.69)	0.0009	0.71
		**Jadad score**					
		4 or 5	483/10087	331/10142	1.47 (1.28, 1.68)	<0.0001	0.62
		<4	133/9159	105/9145	1.26 (0.98, 1.63)	0.07	0.46
	**Aspirin with clopidogrel vs clopidogrel**	**none**	73/3759	22/3781	3.34 (2.08, 5.36)	<0.001	-

We used Egger's test to check for potential publication bias, which showed no evidence of publication bias for the outcomes of major cardiovascular events (P value for Egger's test, 0.674) and myocardial infarction (P value for Egger's test, 0.674). However, we noted evidence of publication bias for stroke (P value for Egger's test, 0.012) and major bleeding events (P value for Egger's test, 0.011). The conclusions were not changed after adjustment for publication bias by the trim and fill method [24].

## Discussion

Recently, evidence from large-scale randomized controlled trials [12], [14] has shown that aspirin plus clopidogrel therapy is not significantly more effective than aspirin alone in reducing the rate of major cardiovascular events. In addition, the harm of life-threatening or major bleeding events has been shown to increase with combined therapy [23]. Our study has shown a possible incremental benefit of adding aspirin to clopidogrel therapy in reducing the harm of major cardiovascular events, myocardial infarction, or stroke. However, the benefits do not outweigh the harms (major bleeding events), when these 2 drugs are given in combination.

The results of our meta-analysis showed that patients assigned aspirin combined with clopidogrel therapy could have reduced harms of major cardiovascular events, myocardial infarction, and stroke, when compared with aspirin therapy alone. However, there was no significant difference between combined therapy and clopidogrel therapy in the relative risk for major cardiovascular events, myocardial infarction, or stroke. The reason for this could be that only 1 trial in such subset. Furthermore, combined therapy significantly increased the harm of major bleeding events, when compared with aspirin or clopidogrel alone. These conclusions are in accordance with the results of some individual trials [12]–[14], which recently reported on the incremental benefit of combined therapy on cardiovascular outcomes and the synergistic effect on major bleeding events. Moreover, we also present the data as absolute risk reduction (ARR) and its confidence intervals to explore clearly that the benefits do not outweigh the harms for combined therapy.

According to the CURE trials [9], dual antiplatelet therapy with clopidogrel plus aspirin had beneficial effects on the harm of major cardiovascular events compared with aspirin therapy alone, but there were also significantly more patients with major bleeding events in the combined therapy group than in the aspirin-alone group. Furthermore, the CHARISMA trial [21] showed that clopidogrel plus aspirin therapy was not significantly more effective than aspirin therapy alone in reducing the rate of major cardiovascular events, myocardial infarction, stroke, and death from vascular disease. Moreover, combined therapy also played an important role in increasing the harm of major bleeding events, whether compared with aspirin or clopidogrel therapy. Our research has defined that benefits could be achieved through the administration of aspirin plus clopidogrel therapy to patients with a history of vascular disease or other vascular-related diseases, in addition, we also noted that adding aspirin to clopidogrel therapy significantly increased the harm of major bleeding events, whether compared with aspirin or clopidogrel therapy. According to ARR and its cobfidence intervals, we easy concluded that combined therapy provided the benefits do not outweigh the harms, which provided by adding aspirin to clopidogrel therapy

Subgroup analysis was also performed, which revealed that high-quality (Jadad score 4 or 5) trials suggested that the harms of major cardiovascular events, myocardial infarction, and stroke were significantly reduced by combined therapy compared with aspirin alone. However, although some beneficial effects on the harm of cardiovascular disease were revealed, aspirin plus clopidogrel therapy also significantly increased the harm of major bleeding events, translating into an increased harm of life-threatening events. The low-quality (Jadad score less than 4) trials suggest no effect of combined therapy on the harm of major cardiovascular events, myocardial infarction, stroke, and major bleeding events. The ACTIVE Trial [13], a trial with long-term follow-up (more than 36 month), suggest that addition of aspirin to clopidogrel could reduce the harm of major vascular events, especially stroke, and increase the harm of major bleeding events. These results were consistent with our study except for the finding that combined therapy reduces the harm of myocardial infarction, only 2 trials included in this subset contributed to this lack of difference, resulting in variation of the conclusion.

In this meta-analysis, benefits were mainly detected in the prevention of major cardiovascular events, myocardial infarction, and stroke. However, significant differences in the harm of major bleeding events were also detected. Furthermore, we noted no clear effect on revascularization or on vascular or nonvascular death. Addition of aspirin to clopidogrel therapy reduced the harm of cardiovascular-related diseases, which may contributed to the reduction in the harm of total mortality and vascular death, these effects may be lessened or balanced by the increased harm of major bleeding events, which contribute to the occurrence of high-life-threatening events.

Previously published relevant randomized controlled trials [9], [11], [21], [22], [25] supported that aspirin and clopidogrel provided uniform benefit. The findings of this meta-analysis also suggested that aspirin plus clopidogrel had a synergistic effect on cardiovascular outcomes and major bleeding events. These findings are credible due to the large volume of data available and the broad range of clinically important features for participants. However, the main limitation of our research was that the result is based on published data, where individual patient data and original data were not available, which limit the capacity to fully explore effects in subgroups. The second limitation of our research was that there was no significant difference between combined aspirin and clopidogrel therapy and clopidogrel therapy alone in reducing the harm in most cardiovascular events except for major bleeding. The reason for the lack of difference may be that only 1 trial provided information for this subset, which limited the investigation of combined therapy compared to clopidogrel therapy alone. Furthermore, clopidogrel therapy alone was found to be superior to aspirin therapy alone in reducing the harm of major cardiovascular events, myocardial infarction, stroke, and vascular death, already demonstrated in the CAORIE trial [10], which may also have contributed to the lack of difference found in our analysis.

In conclusion, the addition of aspirin to clopidogrel therapy protected against major cardiovascular events, myocardial infarction, or stroke, when compared with aspirin therapy alone. It also increased the harm of major bleeding events, whether compared with aspirin or clopidogrel therapy. According to previous findings, the safety profile of drug is of particular importance in public health recommendations for large, apparently disease-free populations. The results of this meta-analysis are promising because we present good evidence that the benefits do not outweigh the harms. Furthermore, our study could help inform personally appropriate judgments about their own use of the combined therapy, and provided evidence to justify general guidelines advocating the routine use of the combined therapy in all patients with high cardiovascular harm factors [26]–[29]. Therefore, we suggest that several factors be improved in future research: (i) The adverse effect events of clinical trials should be recorded and reported normatively, especially for the type of bleeding events, and other non-specific effect adverse reactions should be evaluated in any future trial. (ii) More attention should be given to the role of treatment duration and dosage, and optimal dose and the duration of treatment should be explored. (iii) Addition of aspirin to clopidogrel therapy should be compared not only with aspirin therapy alone but also with clopidogrel therapy alone, which could enable further investigations on the individual effects of aspirin and clopidogrel. (iv) When both aspirin and clopidogrel are administrated to patients, bleeding events should be taken into consideration.

## Supporting Information

Checklist S1 **PRISMA Checklist.**
(DOC)

Protocol S1 **PRISMA Flowchart.**
(DOC)
