# Supplementary material for: Effects of Combined Aspirin and Clopidogrel Therapy on Cardiovascular Outcomes: A Systematic Review and Meta-Analysis
Source: PLoS One. 2012 Feb 13;7(2):e31642. doi: 10.1371/journal.pone.0031642 (PMC3278459; doi:10.1371/journal.pone.0031642)
Supplement: Protocol S1 — PRISMA Flowchart. (DOC) [file pone.0031642.s002.doc]

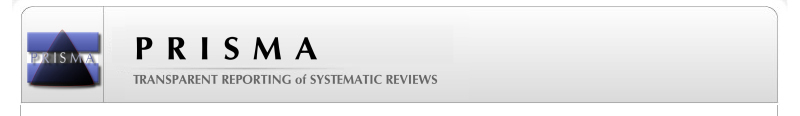
**PRISMA 2009 Flow Diagram**

**Screening**

**Included**

**Eligibility**

**Identification**

7038 Potentially relevant articles identified

6903 Excluded

5280 Irrelevant

556 Were not randomized controlled trails

327 Patients with other therapies

277 Affiliated trials

234 No desirable outcomes

205 No appropriate control

24 Cross-over design

135 Articles retrieved for detailed assessment

128 Excluded

38 Irrelevant

2 Were not randomized controlled trails

32 Patients with other therapies

26 Affiliated trials

17 No desirable outcomes

13 No appropriate control

7 Evaluated both Aspirin and Clopidogrel
